# Supplementary material for: A fault-intrusion-tolerant system and deadline-aware algorithm for scheduling scientific workflow in the cloud
Source: PeerJ Comput Sci. 2021 Nov 2;7:e747. doi: 10.7717/peerj-cs.747 (PMC8576554; doi:10.7717/peerj-cs.747)
Supplement: Supplemental Information 6 [file peerj-cs-07-747-s006.zip › Myproject1.1/docs/org/workflowsim/DatacenterExtended.html]

DatacenterExtended


JavaScript is disabled on your browser.


- Overview
- Package
- Class
- Use
- Tree
- Deprecated
- Index
- Help

- Prev Class
- Next Class

- Frames
- No Frames

- All Classes

- Summary:
- Nested |
- Field |
- Constr |
- Method

- Detail:
- Field |
- Constr |
- Method


org.workflowsim

## Class DatacenterExtended

- java.lang.Object
- - org.cloudbus.cloudsim.core.SimEntity
  - - org.cloudbus.cloudsim.Datacenter
    - - org.workflowsim.DatacenterExtended

- All Implemented Interfaces:
  :   java.lang.Cloneable

  ---

    

  ```
  public class DatacenterExtended
  extends Datacenter
  ```

  DatacenterExtended extends Datacenter so as we can use CondorVM and other
  components

  Since:
  :   WorkflowSim Toolkit 1.0

- - ### Field Summary

    - ### Fields inherited from class org.cloudbus.cloudsim.core.SimEntity

      `FINISHED, HOLDING, RUNNABLE, WAITING`
  - ### Constructor Summary

    Constructors

    Constructor and Description

    `DatacenterExtended(java.lang.String name,
    DatacenterCharacteristics characteristics,
    VmAllocationPolicy vmAllocationPolicy,
    java.util.List<Storage> storageList,
    double schedulingInterval)`
  - ### Method Summary

    Methods

    | Modifier and Type | Method and Description |
    | --- | --- |
    | `protected void` | `checkCloudletCompletion()` Verifies if some cloudlet inside this PowerDatacenter already finished. |
    | `protected void` | `processCloudletSubmit(SimEvent ev, boolean ack)` Processes a Cloudlet submission. |
    | `protected double` | `processDataStageIn(java.util.List<File> requiredFiles, Cloudlet cl)` |
    | `protected void` | `processOtherEvent(SimEvent ev)` Here all the method related to VM requests will be received and forwarded to the related method. |
    | `protected void` | `updateCloudletProcessing()` Updates processing of each cloudlet running in this PowerDatacenter. |

    - ### Methods inherited from class org.cloudbus.cloudsim.Datacenter

      `addFile, contains, contains, getCharacteristics, getHostList, getLastProcessTime, getRegionalCisName, getSchedulingInterval, getStorageList, getVmAllocationPolicy, getVmList, predictFileTransferTime, processCloudlet, processCloudletCancel, processCloudletMove, processCloudletPause, processCloudletResume, processCloudletStatus, processDataAdd, processDataDelete, processEvent, processPingRequest, processVmCreate, processVmDestroy, processVmMigrate, registerOtherEntity, setCharacteristics, setLastProcessTime, setRegionalCisName, setSchedulingInterval, setStorageList, setVmAllocationPolicy, setVmList, shutdownEntity, startEntity`
    - ### Methods inherited from class org.cloudbus.cloudsim.core.SimEntity

      `cancelEvent, clone, getEventBuffer, getId, getName, getNextEvent, getNextEvent, getState, numEventsWaiting, numEventsWaiting, pause, run, schedule, schedule, schedule, schedule, scheduleFirst, scheduleFirst, scheduleFirst, scheduleFirst, scheduleFirstNow, scheduleFirstNow, scheduleFirstNow, scheduleFirstNow, scheduleNow, scheduleNow, scheduleNow, scheduleNow, selectEvent, send, send, send, send, sendNow, sendNow, sendNow, sendNow, setEventBuffer, setId, setState, waitForEvent`
    - ### Methods inherited from class java.lang.Object

      `equals, finalize, getClass, hashCode, notify, notifyAll, toString, wait, wait, wait`

- - ### Constructor Detail


    - #### DatacenterExtended

      ```
      public DatacenterExtended(java.lang.String name,
                        DatacenterCharacteristics characteristics,
                        VmAllocationPolicy vmAllocationPolicy,
                        java.util.List<Storage> storageList,
                        double schedulingInterval)
                         throws java.lang.Exception
      ```

      Throws:
      :   `java.lang.Exception`
  - ### Method Detail


    - #### processOtherEvent

      ```
      protected void processOtherEvent(SimEvent ev)
      ```

      **Description copied from class: `Datacenter`**

      Here all the method related to VM requests will be received and forwarded to the related
      method.

      **Overrides:**
      :   `processOtherEvent` in class `Datacenter`

      Parameters:
      :   `ev` - the received event


    - #### processCloudletSubmit

      ```
      protected void processCloudletSubmit(SimEvent ev,
                               boolean ack)
      ```

      Processes a Cloudlet submission. The cloudlet is actually a job which can
      be cast to org.workflowsim.Job

      **Overrides:**
      :   `processCloudletSubmit` in class `Datacenter`

      Parameters:
      :   `ev` - a SimEvent object
      :   `ack` - an acknowledgement


    - #### processDataStageIn

      ```
      protected double processDataStageIn(java.util.List<File> requiredFiles,
                              Cloudlet cl)
                                   throws java.lang.Exception
      ```

      Throws:
      :   `java.lang.Exception`


    - #### updateCloudletProcessing

      ```
      protected void updateCloudletProcessing()
      ```

      **Description copied from class: `Datacenter`**

      Updates processing of each cloudlet running in this PowerDatacenter. It is necessary because
      Hosts and VirtualMachines are simple objects, not entities. So, they don't receive events and
      updating cloudlets inside them must be called from the outside.

      **Overrides:**
      :   `updateCloudletProcessing` in class `Datacenter`


    - #### checkCloudletCompletion

      ```
      protected void checkCloudletCompletion()
      ```

      Verifies if some cloudlet inside this PowerDatacenter already finished.
      If yes, send it to the User/Broker

      **Overrides:**
      :   `checkCloudletCompletion` in class `Datacenter`


- Overview
- Package
- Class
- Use
- Tree
- Deprecated
- Index
- Help

- Prev Class
- Next Class

- Frames
- No Frames

- All Classes

- Summary:
- Nested |
- Field |
- Constr |
- Method

- Detail:
- Field |
- Constr |
- Method
